# Skeletal Muscle Fatigue in Rats Is More Consistently Related to Increased Inorganic Phosphate Concentration Than Acidosis

**DOI:** 10.1111/apha.70083

**Published:** 2025-07-21

**Authors:** Matthew T. Lewis, Fabio G. Laginestra, Jesse C. Craig, Markus Amann, Russell S. Richardson, Robert W. Wiseman, Ryan M. Broxterman

**Affiliations:** ^1^ Division of Geriatrics, Department of Internal Medicine University of Utah Salt Lake City Utah USA; ^2^ Geriatric Research, Education, and Clinical Center Veterans Affairs Medical Center Salt Lake City Utah USA; ^3^ Department of Physiology Michigan State University East Lansing Michigan USA; ^4^ Department of Anesthesiology University of Utah Salt Lake City Utah USA; ^5^ Department of Nutrition and Integrative Physiology University of Utah Salt Lake City Utah USA; ^6^ Department of Radiology Michigan State University East Lansing Michigan USA

**Keywords:** critical Pi, fatigue, muscle acidity, muscle metabolites, phosphorus magnetic resonance spectroscopy

## Abstract

**Aim:**

Distinguish the relative importance of intramuscular acidosis (hydrogen ion) and inorganic phosphate in skeletal muscle fatigue in vivo in rats.

**Methods:**

We used direct sciatic nerve electrical stimulations to evoke twitches at different frequencies of contraction (0.25‐, 0.50‐, 0.75‐, 1‐, 2‐, and 4‐Hz) in the triceps surae to impose a range of intramuscular metabolic perturbations, quantified by phosphorus nuclear magnetic resonance spectroscopy. Linear mixed‐effects models were used to analyze the relationships between peak twitch force and intramuscular hydrogen ion or inorganic phosphate concentration (as *Z*‐scores) during the protocols that decreased peak twitch force (2‐ and 4‐Hz).

**Results:**

Although intramuscular hydrogen ion and inorganic phosphate concentrations increased with increasing frequencies of contraction, peak twitch force did not begin to decrease until a “threshold” inorganic phosphate concentration was reached. A given hydrogen ion accumulation was associated with a greater decrease in peak twitch force during 4‐Hz compared to 2‐Hz (*β*: −1.19 vs. −0.62, *p* < 0.001). In contrast, the decrease in peak twitch force for a given inorganic phosphate accumulation was not different between 4‐ and 2‐Hz (*β*: −0.89 vs. −0.85, *p* = 0.889).

**Conclusions:**

The inconsistent relationship between the decrease in twitch force and intramuscular hydrogen ion accumulation is not congruent with the primary mechanisms by which acidosis is thought to mediate muscle fatigue. In contrast, the discernible twitch force–inorganic phosphate breakpoint and the consistent relationship between the decrease in twitch force and intramuscular inorganic phosphate accumulation are congruent with the concept of a critical concentration beyond which inorganic phosphate mediates muscle fatigue.

## 1 Introduction

Skeletal muscle, or peripheral, fatigue has been defined as a rapidly developing, reversible decline in the contractile performance of the muscle fibers [1]. During intense contractions, muscle fatigue is routinely quantified by the decrease in maximal force production for a given stimulus and is mechanistically linked to intramuscular biochemical changes [2, 3]. There is ample evidence that hydrogen ion (H^+^; i.e., acidity) and/or inorganic phosphate (Pi) in the muscle are the primary biochemical factors that mediate muscle fatigue [2]. Indeed, muscle fatigue development is strongly related to intramuscular H^+^ concentration [4, 5, 6, 7], and H^+^ has been reported to directly decrease force production by reducing the force per cross‐bridge [8] or myofibrillar calcium (Ca^2+^) sensitivity [9]. Likewise, intramuscular Pi concentration is related to muscle fatigue development [2, 10, 11] and has been shown to decrease force production by reducing the number of bound cross‐bridges [12], myofibrillar Ca^2+^ sensitivity [13, 14], or Ca^2+^ release from the sarcoplasmic reticulum [2, 15]. While both H^+^ and Pi have been shown to mediate muscle fatigue ex vivo, it has been difficult to distinguish their relative importance in the development of skeletal muscle fatigue in vivo.

The strength and consistency of the in vivo relationships between muscle fatigue and intramuscular H^+^ and Pi are fundamental to revealing the relative importance of each metabolite. In vivo and in situ, there is a strong linear relationship between muscle fatigue and intramuscular H^+^ concentration during isometric contractions [16, 17, 18]. The consistency of this relationship, however, is equivocal because it can be altered or abolished with different interventions [16, 19, 20], which questions the relative importance of H^+^ in mediating muscle fatigue in vivo. Similarly, the relative importance of Pi in mediating muscle fatigue in vivo can be questioned because of the evidence that muscle fatigue does not develop until intramuscular Pi reaches a relatively high concentration during isometric contractions in vivo or in situ [16, 21, 22, 23]. Importantly, although underappreciated, such a non‐linear relationship between muscle fatigue development and intramuscular Pi accumulation is congruent with ex vivo evidence that a certain myoplasmic Pi concentration is required for Pi to impair Ca^2+^ release from the sarcoplasmic reticulum [11, 24, 25, 26, 27]. Yet these previous studies did not systematically analyze an in vivo “threshold” Pi concentration [16, 21, 22, 23]. In a first attempt to incorporate the abovementioned concepts in vivo, we detected a “threshold” intramuscular Pi concentration above which the rate of decrease in twitch force drastically increased (i.e., twitch force‐Pi breakpoint) in humans during fatiguing quadriceps isometric contractions [18]. We also detected strong linear relationships between the decrease in twitch force and both H^+^ and Pi (above the twitch force‐Pi breakpoint) accumulation, but the relationship for H^+^ was not consistent across the two consecutive contraction bouts [18]. Unfortunately, the precision of the twitch force–Pi breakpoint in our previous study was limited by relatively few data points prior to the breakpoint. Overall, it is important to assess the relationships between the decrease in twitch force and H^+^ or Pi concentrations across a wide range of metabolic rates in vivo to improve the precision of the twitch force‐Pi breakpoint and to determine the consistency of the relationships between each metabolite and muscle fatigue.

Accordingly, the aims of this study were to (1) quantify the twitch force–Pi breakpoint and (2) determine the consistency of the relationships between the decrease in twitch force and H^+^ and Pi concentration during bouts of fatiguing isometric muscle contractions. These aims were attained by concurrent quantification of muscle peak twitch force and intramuscular metabolite concentrations during bouts of electrically evoked muscle twitches across a wide range of frequencies in rats. We hypothesized (1) a consistent relationship between the decrease in twitch force and intramuscular Pi accumulation beyond the twitch force–Pi breakpoint and (2) an inconsistent relationship between the decrease in twitch force and intramuscular H^+^ accumulation.

## 2 Methods

### 2.1 Animals

Male Wistar rats (*n* = 7) from Charles River Laboratories (Wilmington, MA) were studied at 15–17 weeks of age. Animals were housed 2–3 per cage in a temperature‐controlled room and provided food (NIH‐31M: 23% calories from protein, 18% calories from fat, and 59% calories from carbohydrate) and water *ad libitum*. Animals were maintained on a 12:12‐h light–dark cycle. All procedures were performed at Michigan State University and approved by the Michigan State University Institutional Animal Care and Use Committee and complied with The American Physiological Society's “Guiding Principles in the Care and Use of Animals.” All the submitted material is in conformance with good publishing practice in physiology [28]. Of note, some of the data acquired from these animals have been presented previously, albeit with a different experimental question [29].

### 2.2 Experimental Protocol

**FIGURE 1:**
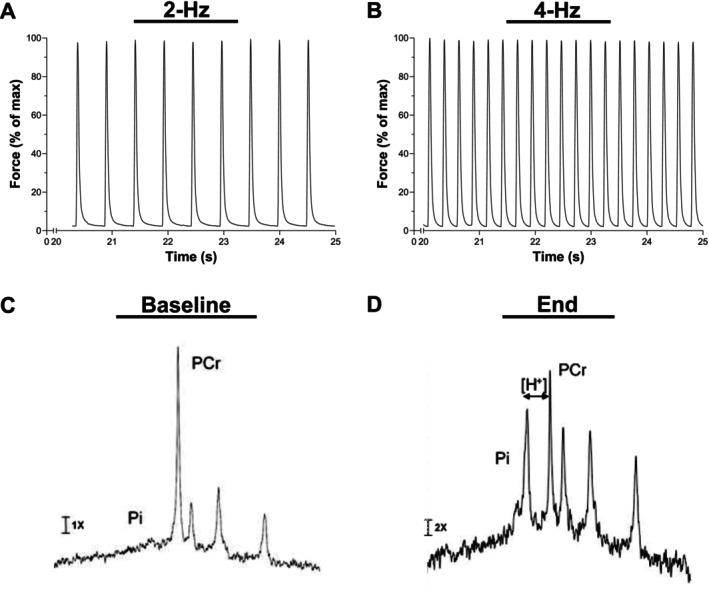
Representative force recordings during 2‐Hz (Panel A) and 4‐Hz (Panel B) frequencies of contraction, as well as representative phosphorus nuclear magnetic resonance spectroscopy spectra at baseline (Panel C) and end of protocol (Panel D). H^+^, hydrogen ion; PCr, phosphocreatine; Pi, inorganic phosphate.

Animals were fasted for 12‐h overnight preceding experimental days. Surgical procedures began with animals anesthetized at 3%–5% isoflurane in 100% O_2_, depth of anesthesia ensured by toe pinch, and subsequently maintained at 1%–2% isoflurane throughout experimentation. Respiration rate and rectal temperature were monitored and maintained at 45–60 breaths/min and 36°C–37°C, respectively. The surgical preparation was performed as previously described [29, 30, 31] for direct nerve electrical stimulation to evoke isometric contraction of the entire *triceps surae* (i.e., gastrocnemius, plantaris, soleus) within a surface coil for ^31^P‐MRS. In brief, using a custom‐built nuclear magnetic resonance (NMR) probe, the knee was fixed to a brass post with a tungsten pin through the head of the femur, and the Achilles tendon was tied to an isometric force transducer. This orientation positioned the superficial gastrocnemius muscle over a 1.7 cm diameter surface coil wound from two turns of copper wire. Optimal length was determined by progressively stretching the muscle until electrical stimulation no longer increased peak twitch force, and this muscle length was maintained throughout the protocol to ensure complete muscle activation [32, 33]. Muscle twitches were evoked by electrical nerve stimulations (2–10 V, 2 ms duration) applied via bipolar platinum electrodes on the sciatic nerve at 120% of the voltage that elicited maximal twitch force. Muscle twitches were performed in random order at frequencies of 0.25‐, 0.5‐, 0.75‐, 1‐, 2‐, and 4‐Hz for 4.8 min to simulate a range of aerobic/anaerobic workloads. Individual twitches were quantified for peak force using the *event analysis* algorithm [34], which runs in MATLAB (Mathworks Inc., Natick, MA, USA), and binned by averaging over 24 s to match the time resolution of the phosphorus spectra. Representative force recordings for 2‐ and 4‐Hz are presented in Figure 1A,B. At the end of the experiment, the animal was euthanized by an overdose of isoflurane, and all lower leg muscles were removed and weighed.

Phosphorus NMR spectra were acquired in the Fourier transform mode and tuned to detect ^31^P at 161.8 MHz within a Bruker AM400 wide‐bore spectrometer (9.4T, 7.4 cm vertical bore magnet) housed in the Department of Physiology at Michigan State University. The initial signal was optimized by tuning the coil with the animal in place, and the magnetic field was shimmed using the available proton signal from water. Phosphorus spectra were optimized based on the Ernst angle for signal‐to‐noise ratio [35] determined at 4.5 μs pulse width and 1.5 s repetition time. Spectra were continuously acquired over 24 s intervals (8012‐Hz sweep width; 4096 complex points; 1.5 s interpulse interval; 16 averages) during 144 s of rest (6 spectra), 288 s of stimulation (12 spectra), and 432 s of passive recovery (18 spectra). At least 20 min of rest was permitted between muscle contraction bouts, and metabolites were verified to have returned to the initial resting baseline values before subsequent stimulation bouts. NMR signals were corrected for partial saturation by acquiring 64 free induction decays (FIDs) before stimulation under the experimental conditions and at 5 × *T*1 (where *T*1 is the spin–lattice relaxation time) for phosphocreatine (PCr) at 9.4T (~3 s, thus repetition time = 15 s).

Summed FIDs were multiplied by a 30‐Hz exponential line broadening, matched to the average line width acquired for PCr, before Fourier transformation. ^31^P signal integrals were computed using jMRUI AMARES fitting software [36]. Metabolite concentrations were determined from PCr to ATP ratios after normalizing to total phosphate integral and normalized to absolute ATP content quantified by high‐performance liquid chromatography [37, 38] as previously reported [29]. Representative spectra showing Pi, PCr, and the three phosphate peaks of ATP at baseline and the end of the protocol are presented in Figure 1C,D. Intracellular [H^+^] was estimated from the chemical shift of Pi relative to PCr (ΔPPM) [39, 40]:

$$H^{+}=10^{-\left(6.75+\log\left(\frac{3.37-\text{ΔPPM}}{\text{ΔPPM}-5.63}\right)\right)}$$

When recovery of intramuscular metabolites or force (despite continued electrical stimulation) was evident, subsequent data were excluded from analysis.

### 2.3 Statistics

The twitch force/Pi breakpoint was determined by fitting peak twitch force (% of maximum) vs. Pi with a two‐segment piecewise linear function to all data across all frequencies of contraction within each animal:

$$\left(\text{1st segment}\right)\;{\text{twitch force}}_{1}=m_{1}\times \mathrm{Pi}+b_{1},\text{for}\;\mathrm{Pi}<{\mathrm{Pi}}_{\mathrm{BP}};$$

$$\left(\text{2nd segment}\right)\;{\text{twitch force}}_{2}=\left(m_{1}\times {\mathrm{Pi}}_{\mathrm{BP}}+b_{1}\right)+m_{2}\;\left(\mathrm{Pi}–{\mathrm{Pi}}_{\mathrm{BP}}\right),\text{for}\;\mathrm{Pi}\geq {\mathrm{Pi}}_{\mathrm{BP}};$$

where *b*_1_ and *m*_1_ are, respectively, the *y*‐intercept and slope of the first linear segment, Pi_BP_ is the Pi concentration at the breakpoint between the two segments (i.e., twitch force/Pi breakpoint), and *m*_2_ is the slope of the second linear segment (GraphPad Prism version 10.0.0, GraphPad Software, Boston, MA, USA). The two‐segment piecewise linear function was also fit to peak twitch force versus H^+^ to test for a breakpoint. The goodness of fit was compared between the two‐segment linear function and a linear function using the extra sum‐of‐squares *F* test to determine which fit these data the best.

Linear mixed‐effects models were used to analyze the relationships between peak twitch force and [H^+^] or [Pi] (Stata *mixed*, Stata 17, StataCorp LLC, College Station, TX, USA). The twitch frequencies with a significant decrease in peak force (% of maximum) over time, determined by one‐way ANOVAs, were included in the models. Within these twitch frequencies, data were included from the onset of muscle twitches until peak twitch force reached a nadir (< 3% point‐to‐point change in twitch force relative to maximum). These steps focus the assessments on when twitch force is decreasing and minimize the leveraging impact of a plateau in peak twitch force on the linear regression analyses. Pi concentrations less than the twitch force/Pi breakpoint were not included in the model based on evidence that Pi does not induce muscle fatigue until attaining a certain concentration (i.e., Ca^2+^‐Pi precipitation in sarcoplasmic reticulum). The linear mixed‐effects models consisted of a dependent variable (peak twitch force as a % of maximum), independent variables (H^+^ or Pi × twitch frequency interaction) as fixed effects, and random effects for each animal (intercept) to account for repeated measures within animals. The standardized (*Z*‐score) dependent (peak twitch force % of maximum) and independent (H^+^ and Pi) variables were used for the linear mixed‐effects models to determine the standardized regression coefficients (*β*) and allow more appropriate comparison of the fixed effects across models. The *Z*‐score was calculated by subtracting the group mean from individual measurements and then dividing by the group standard deviation, thereby normalizing the data to a common scale and facilitating model comparisons. Post hoc *β*‐coefficient comparisons of model slopes and y‐intercepts between twitch frequencies within each model (H^+^ or Pi) were corrected using the Holm‐Bonferroni method. Statistical significance was set at *p* < 0.05. Linear mixed‐effects model data are presented as *β*‐coefficient, 95% confidence interval (CI), and *p*‐value. All other data are presented as individual or mean ± standard deviation.

## 3 Results

### 3.1 Animal Characteristics, Muscle Force Production, and Metabolism

**FIGURE 2:**
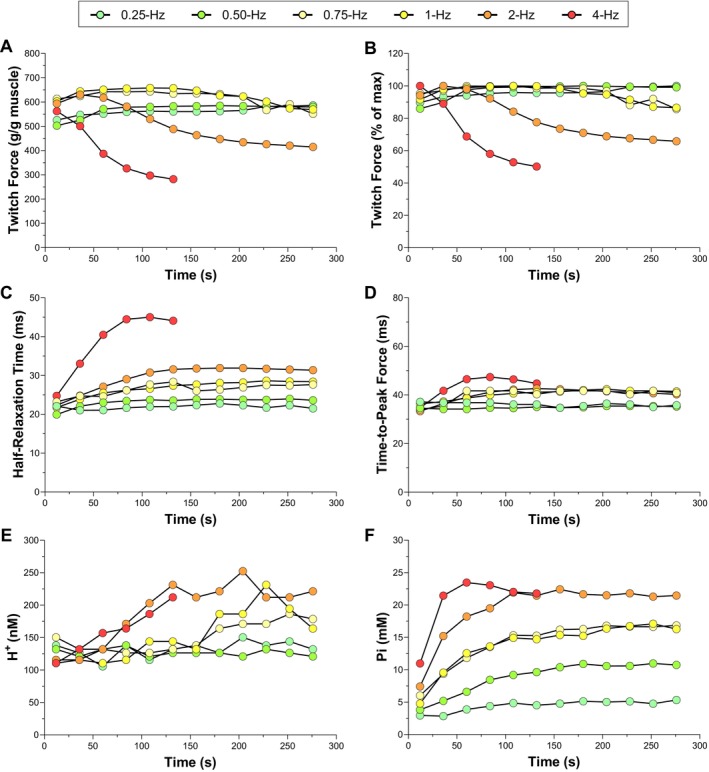
Absolute (Panel A) and relative (Panel B) peak twitch force, twitch force half‐relaxation time (Panel C) and time‐to‐peak force (Panel D), intramuscular hydrogen ion (H^+^) (Panel E), and inorganic phosphate (Pi) (Panel F) during 0.25‐, 0.5‐, 0.75‐, 1‐, 2‐, and 4‐Hz frequencies of contraction in a representative rat. Data for the 4‐Hz frequency of contraction are presented only until 2.4 min (see *Methods* for details).

Animal body mass was 459 ± 16 g, and *triceps surae* mass was 2.84 ± 0.19 g. Absolute and relative values of muscle peak twitch force over time for all frequencies of contraction are presented for a representative rat in Figure 2A,B. There was no decrease in peak twitch force over time for the 0.25‐, 0.5‐, 0.75‐, and 1‐Hz frequencies of contraction (all *p* > 0.062). The decrease in peak twitch force over time was significant for 2‐ and 4‐Hz, with peak twitch force dropping to ~70% and ~43% of maximum, respectively. Twitch force half‐relaxation time and time‐to‐peak are presented over time for all frequencies of contraction for the representative rat in Figure 2C,D. There was a significant time effect for twitch force half‐relaxation time within each frequency of contraction (all *p* < 0.039). Compared to the first measurement, the half‐relaxation time at the end of each protocol was increased by 1%, 18%, 9%, 31%, 34%, and 55% within the 0.25‐, 0.5‐, 0.75‐, 1‐, 2‐, and 4‐Hz frequencies of contraction, respectively. There was no significant time effect for time‐to‐peak twitch force within any frequencies of contraction (all *p* > 0.050). Intramuscular H^+^ and Pi concentrations over time for all frequencies of contraction are presented for the representative rat in Figure 2E,F. Although H^+^ concentration was used for all analyses, the pH values at the end of each protocol are reported here to indicate the extent of acidosis: 6.90, 6.93, 6.87, 6.77, 6.70, and 6.71 for the 0.25‐, 0.5‐, 0.75‐, 1‐, 2‐, and 4‐Hz frequencies of contraction, respectively.

### 3.2 Twitch Force/Pi or H^+^ Breakpoint

**FIGURE 3:**
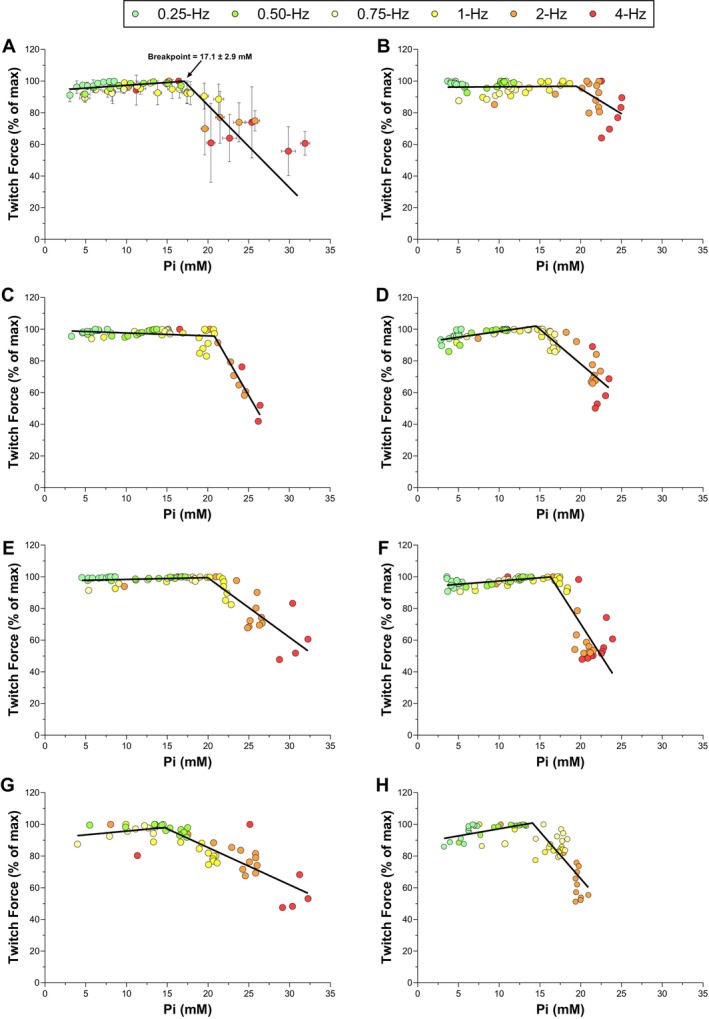
The relationship between relative peak twitch force and intramuscular inorganic phosphate (Pi) during electrically evoked contractions at 0.25‐, 0.5‐, 0.75‐, 1‐, 2‐, and 4‐Hz. Group mean data (Panel A) and data for each rat individually (Panels B–H) are presented. The group data (*n* = 7, except *n* = 6 for 0.25‐ and 4‐Hz) are presented as mean ± SD. One rat did not have data for 0.25‐Hz (Panel G), and one rat did not have data for 4‐Hz (Panel H) because of technical issues. Two‐segment piecewise linear functions (solid lines) were fit to the individual data (Panels B–H) to determine the twitch force–Pi breakpoint, which occurred at [Pi] = 17.1 ± 2.9 mM (Panel A). The two‐segment linear function shown with the group data (Panel A) represents the average relationship and was not generated by fitting the function to the group mean data.

**FIGURE 4:**
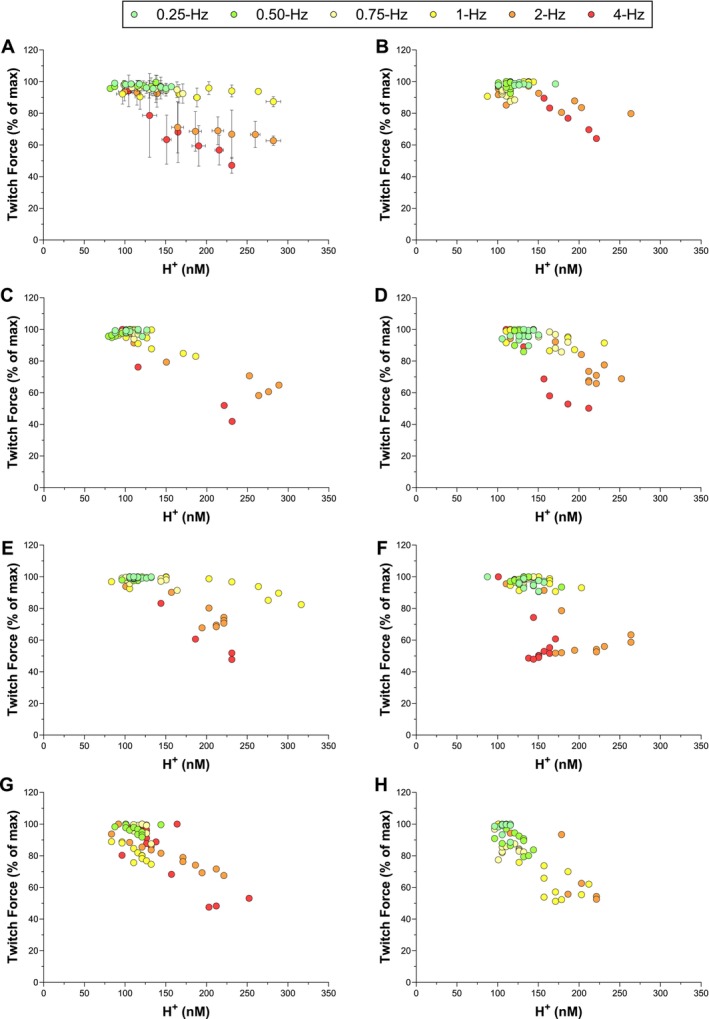
The relationship between relative peak twitch force and intramuscular hydrogen ion (H^+^) during electrically evoked contractions at 0.25‐, 0.5‐, 0.75‐, 1‐, 2‐, and 4‐Hz. Group mean data (Panel A) and data for each rat individually (Panels B—H) are presented. The group data (*n* = 7, except *n* = 6 for 0.25‐ and 4‐Hz) are presented as mean ± SD. One rat did not have data for 0.25‐Hz (Panel G), and one rat did not have data for 4‐Hz (Panel H) because of technical issues. Two‐segment piecewise linear functions (with a negative slope of the second segment) were fit to the individual data to test for a twitch force–H^+^ breakpoint. This piecewise function provided a better fit than a simple linear function in only two rats (Panels B and C).

Peak twitch force (% of maximum) is plotted versus Pi concentration for all frequencies of contraction in Figure 3. The mean data are presented in Figure 3A, while panels B–H present the data for each rat separately. A two‐segment linear function (with a negative slope of the second segment) fit these data significantly better than a linear function in all 7 rats (all *p* < 0.001). The twitch force/Pi breakpoint (i.e., Pi_BP_ in the regression model) was detectable for all rats and was found at [Pi] = 17.1 ± 2.9 mM (*r* ^2^ = 0.73 ± 0.17, *p* < 0.05). Therefore, all the Pi values below this concentration (i.e., when peak twitch force was still stable) were excluded from the linear mixed‐effects models on an individual basis. Peak twitch force (% of maximum) is plotted versus H^+^ concentration for all frequencies of contraction in Figure 4. The mean data are presented in Figure 4A, while panels B–H present the data for each rat separately. These data were better fit by the two‐segment linear function (with a negative slope of the second segment) than a linear function in only 2 out of the 7 rats (2 rats are in Figure 4B,C). Thus, there was no consistent evidence of a twitch force/H^+^ breakpoint and therefore no H^+^ data were excluded from the linear mixed‐effects models.

### 3.3 Twitch Force and H^+^ or Pi Relationships

**FIGURE 5:**
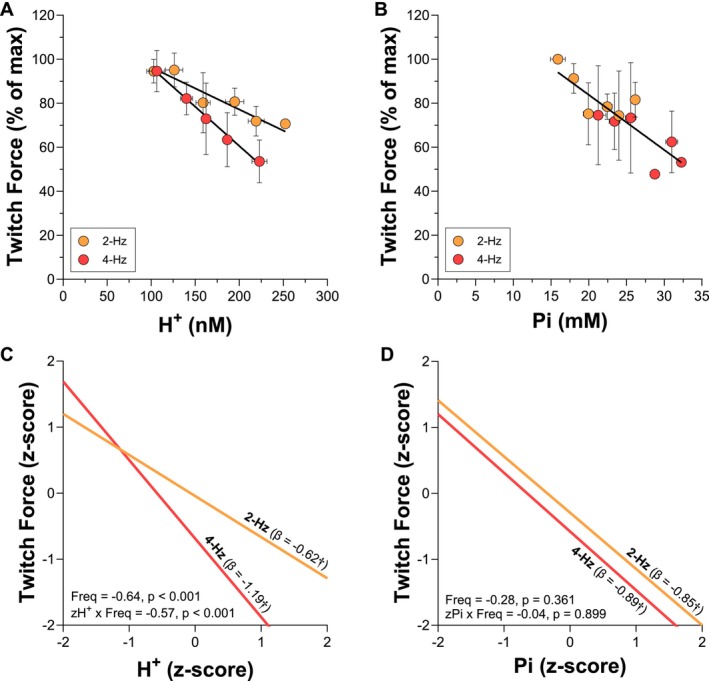
The relationship between relative peak twitch force and intramuscular hydrogen ion (H^+^) and inorganic phosphate (Pi) during 2‐ and 4‐Hz frequencies of contraction is shown as raw concentrations (Panels A and B) and *Z*‐scores (Panels C and D). Group mean data (Panels A and B) are presented for illustrative purposes only and do not accurately reflect the individual, within‐animal relationships. ^†^Indicates that the slope of the model was significantly different from zero (*p* < 0.001). For peak twitch force versus H^+^ (Panel C), both the slopes and y‐intercepts were significantly different between 2‐ and 4‐Hz, indicating stimulation frequency‐dependent differences in the relationship between the decrease in twitch force and the accumulation of H^+^. For peak twitch force versus Pi (Panel D), neither the slopes nor y‐intercepts were significantly different between 2‐ and 4‐Hz, indicating a consistent relationship between the decrease in twitch force and the accumulation of Pi across stimulation frequencies.

The group mean data and linear regressions are presented for illustrative purposes in Figure 5A,B. However, it is important to note that these summaries do not accurately represent the within‐animal relationships, which are more appropriately modeled using linear mixed‐effects analyses. The 2‐ and 4‐Hz frequencies of contraction were used in the linear mixed‐effects models because there was a significant decrease in peak twitch force over time during these (both *p* < 0.004). The linear mixed‐effects model analyses of the relationship between peak twitch force and H^+^ or Pi are presented in Figure 5C,D. The slopes of the relationships between peak twitch force and H^+^ were significantly different from zero for 2‐Hz (*β* = −0.62, CI = −0.77 to −0.47, *p* < 0.001) and 4‐Hz (*β* = −1.19, CI = −1.40 to −0.98, *p* < 0.001). The y‐intercept of the relationship between peak twitch force and H^+^ was significantly different from zero for 4‐Hz (*β* = −0.69, CI: −1.03 to −0.36, *p* < 0.001) but not 2‐Hz (*β* = −0.05, CI = −0.35 to 0.26, *p* = 0.758). There was a significant interaction between H^+^ and frequency of contraction such that the slope of the relationship between peak twitch force and H^+^ was significantly greater for 4‐Hz compared with 2‐Hz (H^+^ × Frequency *β* = −0.57, CI = −0.83 to −0.31, *p* < 0.001) and the y‐intercept was significantly lesser for 4‐ compared with 2‐Hz (Frequency *β* = −0.64, CI = −0.91 to −0.38, *p* < 0.001) (Figure 5C). Similar to H^+^, the slopes of the relationships between peak twitch force and Pi were significantly different from zero for 2‐Hz (*β* = −0.85, CI = −1.36 to −0.35, *p* = 0.001) and 4‐Hz (*β* = −0.89, CI = −1.32 to −0.46, *p* < 0.001). The y‐intercepts of the relationships between peak twitch force and Pi, contrary to H^+^, were not significantly different from zero for 2‐Hz (*β* = −0.30, CI = −0.90 to 0.30, *p* = 0.331) and 4‐Hz (*β* = −0.58, CI = −1.36 to 0.21, *p* = 0.296). Also, contrary to H^+^, there was no significant interaction between Pi and frequency of contraction such that the slopes of the relationship between peak twitch force and Pi were not different between 4‐ and 2‐Hz (Pi × Frequency *β* = −0.04, CI: −0.60 to 0.53, *p* = 0.899) or *y*‐intercept (Frequency *β* = −0.28, CI: −0.88 to 0.32, *p* = 0.361) (Figure 5D).

## 4 Discussion

Intramuscular H^+^ and Pi have long been studied as the primary biochemical mediators of skeletal muscle fatigue, but distinguishing their relative importance in vivo remains difficult. In the present study, a range of rates and magnitudes of H^+^ and Pi accumulation in rat muscle were imposed by electrically evoked muscle twitches at different frequencies of contraction (0.25‐, 0.50‐, 0.75‐, 1‐, 2‐, and 4‐Hz). In accordance with our first hypothesis, the decrease in twitch force was negligible until a “threshold” intramuscular Pi concentration was reached across the different frequencies of contraction (i.e., twitch force–Pi breakpoint). Above this twitch force–Pi breakpoint, the magnitude of decrease in twitch force for a given intramuscular Pi accumulation was not different between the 2‐ and 4‐Hz frequencies of contraction. Supporting our second hypothesis, there was a two‐fold difference in the magnitude of decrease in twitch force for a given accumulation of intramuscular H^+^ between the 2‐ and 4‐Hz frequencies of contraction. While the current study did not directly assess the causal role of H^+^ or Pi in the development of muscle fatigue, the discernible twitch force–Pi breakpoint and consistent relationship between the decrease in twitch force and intramuscular Pi accumulation are congruent with the concept of a critical Pi concentration beyond which Pi mediates muscle fatigue. In contrast, the inconsistent relationship between the decrease in twitch force and intramuscular H^+^ accumulation is not congruent with the primary mechanisms by which acidosis is thought to mediate muscle fatigue.

### 4.1 Muscle Fatigue Measured by Twitch Force

In this study, peak twitch force either did not decrease (0.25‐, 0.5‐, 0.75‐, 1‐Hz) or decreased later in the stimulation protocol (2‐ and 4‐Hz). Twitch force, as with all other indices of submaximal force, is influenced by factors beyond muscle fatigue per se, which can mask the true extent of fatigue during the initial portions of the protocol. Indeed, studies employing tetanic muscle contractions consistently demonstrate a small but significant decline in maximal force in the early phase of the protocol (i.e., Phase I), likely attributable to direct effects of accumulating metabolites on actomyosin cross‐bridge cycling or altered Ca^2+^ sensitivity [11, 15, 41]. In the current study, this early phase of fatigue may not have been evident because of opposing mechanisms that coexist with muscle fatigue (e.g., increased Ca^2+^ release or sub‐tetanic force potentiation) [42]. Supporting the presence of early fatigue, twitch force half‐relaxation time, another established indicator of muscle fatigue, was prolonged during the early portion in nearly all stimulation protocols, indicating that some degree of muscle fatigue likely developed despite no discernible decrease in peak twitch force. Importantly, the similarity between the twitch force/Pi breakpoint and the onset of late‐phase fatigue observed in tetanic contraction studies (i.e., Phase 3) strongly supports that the twitch force/Pi breakpoint reflects the point at which sarcoplasmic reticulum Ca^2+^ release becomes limiting to force production [43].

### 4.2 Muscle Fatigue Related to Intramuscular Pi

In the current study, intramuscular Pi attained submaximal concentrations without significant decreases in twitch force during the 0.25‐, 0.50‐, 0.75‐, and 1‐Hz frequencies of contraction, which aligns with the previously reported oxidative capacity in rat gastrocnemius muscle [44]. In contrast, intramuscular Pi accumulated to near‐maximal concentrations with substantial decreases in twitch force during the 2‐ and 4‐Hz frequencies of contraction (Figures 2 and 3). Critically, decreases in twitch force were negligible until a “threshold” intramuscular Pi concentration was reached across the different frequencies of contraction, which we defined as the twitch force–Pi breakpoint (Figure 3). This detectable breakpoint in rats is consistent with a recent study in which we found evidence, although with limited precision, of a twitch force–Pi breakpoint in humans [18]. There are several additional studies in which muscle fatigue did not develop until intramuscular Pi reached a relatively high concentration during isometric contractions in muscle in vivo and in situ, but this was not systematically analyzed [16, 21, 22, 23]. The current study estimated the twitch force‐Pi breakpoint with improved precision by imposing a range of rates and magnitudes of Pi accumulation that spanned the oxidative and non‐oxidative range in rat muscle. Moreover, accuracy was further improved by normalizing the phosphate concentrations measured using ^31^P‐MRS to the absolute ATP concentrations quantified ex vivo. Although the accuracy of the Pi concentrations may be limited by standardization procedures in other studies (e.g., 8.2 mM ATP), the suggested intramuscular Pi concentration at the twitch force‐Pi breakpoint was between ~15–20 mM in these previous studies and the current study [16, 18, 21, 22, 23]. Additional studies are needed to discern the absolute Pi concentrations at the twitch force‐Pi breakpoint across species, muscle types, and diseases. It is important to note that the twitch force‐Pi breakpoint is consistent with the theoretical concept of a “critical” Pi concentration (equivalent to the twitch force–Pi breakpoint) that distinguishes the metabolic rate above which Pi progressively increases to near‐maximal values (i.e., critical metabolic rate or critical power) [45].

In the current study, there was a strong linear relationship between the decrease in twitch force and intramuscular Pi when Pi concentration exceeded the twitch force–Pi breakpoint during the 2‐ and 4‐Hz frequencies of contraction (Figures 3 and 5D). Moreover, the slope and y‐intercept of this relationship did not change across 2‐ and 4‐Hz frequencies of contraction within the same rat, despite different rates and magnitudes of intramuscular Pi accumulation. These findings are in agreement with the consistent relationship between the decrease in twitch force and intramuscular Pi accumulation in humans during consecutive voluntary quadriceps isometric contraction protocols [18]. Together, these data support an intramuscular Pi concentration above which muscle fatigue begins to develop (i.e., twitch force–Pi breakpoint). Once this breakpoint is exceeded, there is a strong linear relationship between the decrease in twitch force and intramuscular Pi accumulation that does not vary across fatiguing protocols within the same animal or person.

If intramuscular Pi is a primary mediator of muscle fatigue, then the mechanism of action must be consistent with the existence of a twitch force–Pi breakpoint (Figure 3) [18]. Previous studies in skinned fibers have demonstrated that Pi can reduce myofibrillar force production [46] and Ca^2+^ sensitivity [13], which may mediate the relatively small muscle fatigue development when myoplasmic Ca^2+^ is high. Yet these mechanisms are not likely to explain the majority of muscle fatigue that develops when myoplasmic Ca^2+^ concentration decreases during isometric contractions. Indeed, there is evidence that Pi primarily reduces maximal force production by impairing Ca^2+^ release from the sarcoplasmic reticulum and preventing Ca^2+^ binding to troponin in intact muscle fibers ex vivo, where the fibers maintain their in vivo reliance on excitation‐contraction coupling and determination of biochemical changes (e.g., Ca^2+^ and metabolites) [2, 26, 47, 48]. Several previous studies using intact muscle fibers demonstrated that maximal force production and myoplasmic Ca^+2^ decreased concomitantly during isometric contractions, caffeine‐induced restoration of myoplasmic Ca^2+^ restored maximal force production, intramuscular Pi impaired maximal force production, and intramuscular H^+^ had no effect on maximal force production during the isometric contractions [2, 15, 47, 48]. Additionally, there is evidence that Pi enters the sarcoplasmic reticulum through an ATP‐sensitive channel where it precipitates with Ca^2+^ and impairs Ca^2+^ release in skinned muscle fibers with an intact sarcoplasmic reticulum and in single channels [24, 25, 27, 49, 50]. Critically, there was a certain Pi concentration above which Pi entered the sarcoplasmic reticulum in both a concentration‐ and time‐dependent manner [24, 25, 27, 49, 50]. In intact muscle fibers, injecting Pi into the fiber decreased maximal force production and Ca^2+^ release from the sarcoplasmic reticulum [11]. Thus, the in vivo twitch force‐Pi breakpoint is consistent with the mechanism by which Pi concentration has reached a level where Pi begins to enter into the sarcoplasmic reticulum, and the development of muscle fatigue is consistent with the mechanism by which Ca–Pi precipitation impairs Ca^2+^ release from the sarcoplasmic reticulum.

### 4.3 Muscle Fatigue Related to Intramuscular H^+^

In the current study, there was a strong linear relationship between the decrease in twitch force and intramuscular H^+^ accumulation during the 2‐ and 4‐Hz frequencies of contraction (Figures 4 and 5C). Yet the slopes of these relationships differed such that the same change in intramuscular H^+^ was associated with approximately double the rate of decrease in twitch force during the 4‐Hz compared to the 2‐Hz frequencies of contraction (Figure 5C). Furthermore, the y‐intercept of this relationship differed between 4‐ and 2‐Hz frequencies of contraction, such that a given H^+^ concentration was associated with different magnitudes of decrease in twitch force within the same muscle. These results are congruent with a recent study in humans where the y‐intercept of the relationship between the decrease in twitch force and H^+^ changed between two sequential quadriceps isometric contraction protocols [18]. Overall, these data support that intramuscular H^+^ concentration can be strongly correlated to the decrease in twitch force, but the varied relationships across protocols within the same animal or person add to the uncertainty of the relative importance of H^+^ in mediating muscle fatigue in vivo.

The relationship between muscle acidity (i.e., H^+^) and muscle fatigue was proposed as early as 1865 [51] and has been studied extensively ever since. The development of muscle fatigue is often strongly related to the accumulation of intramuscular H^+^ [6]. Nelson and Fitts [9] demonstrated that intramuscular H^+^ (pH 6.2) decreased myofibrillar Ca^2+^ sensitivity in skinned muscle fibers ex vivo. The decrease in myofibrillar Ca^2+^ sensitivity appears to arise from either H^+^ decreasing troponin C binding affinity for Ca^2+^ or decreasing the binding affinity between troponin I and troponin C [9, 52, 53]. While intramuscular H^+^ has also been demonstrated to reduce shortening velocity and peak power in skinned muscle fibers ex vivo [5], which appears to result from alterations in ADP at the level of the cross‐bridge [54], its impact on maximal isometric force production at physiological temperatures is relatively small [5, 55]. There is also evidence that H^+^ may actually protect against muscle fatigue within the physiological pH range [20, 56]. The evidence that H^+^, in addition to Pi, mediates muscle fatigue is primarily from studies using skinned muscle fibers or isolated actin‐myosin molecules. While these techniques are certainly important, fixing Ca^2+^ concentrations prevents assessing the role of changes in myoplasmic Ca^2+^ concentration in mediating muscle fatigue, effectively eliminating the evaluation of the impact of Pi by its postulated role in intact muscle fibers. Indeed, the mechanisms by which intramuscular H^+^ is hypothesized to induce muscle fatigue are not congruent with the inconsistent relationship between the decrease in twitch force and intramuscular H^+^ accumulation in our current and previous studies [18]. The altered relationship between muscle contraction protocols within the same animal supports that either the “sensitivity” of the muscle to H^+^ changes or that H^+^ was not the primary factor mediating the decrease in twitch force. The current findings do not categorically exclude H^+^ as a mediator of muscle fatigue development, nor do they provide insight into potential indirect effects H^+^ may exert on fatigue through mechanisms such as muscle blood flow, enzyme function, and ion transport. However, the current findings do contribute to the growing body of evidence from intact muscle and isolated muscle fibers supporting that muscle fatigue development can occur independently of intramuscular H^+^ accumulation during isometric contractions [15, 16, 19, 41]. Overall, the inconsistent relationship between the decrease in twitch force and intramuscular H^+^ accumulation found in the current study supports that muscle acidosis was likely not the primary factor mediating muscle fatigue in this study.

### 4.4 Experimental Considerations

The current experimental protocol was specifically designed to isolate and focus on the relationship between intramuscular metabolites and the decrease in twitch force during in vivo muscle contractions. To enhance the signal‐to‐noise ratio of the MRS data, we averaged 16 spectra over each 24‐s period. While this approach significantly improves data quality, it necessarily limits temporal resolution and precludes high‐frequency assessments (e.g., second‐by‐second). Additionally, the MRS technique provides a spatially averaged measurement of the muscle and cannot resolve metabolite concentrations within specific subcellular compartments, such as the sarcoplasmic reticulum, or within individual fiber types. By design, the combined mechanical and biochemical assessments provide an integrated in vivo evaluation of muscle function under physiologically relevant conditions. We acknowledge that the findings must be interpreted within this integrated context rather than as uniform conditions, given that the measured force arises from multiple muscles comprising the triceps surae, each containing heterogeneous fiber types and metabolic profiles, and considering the differences in spatial resolution between the volume‐averaged mechanical measurements and the more localized MRS measurements. While these factors limit our ability to directly link observed responses to specific fiber types and mechanisms, this inherent trade‐off enables characterization of complex, system‐level muscle dynamics that are difficult to interrogate with isolated or ex vivo techniques, thereby offering novel insights into muscle physiology. While electrical stimulation precludes the impact and assessment of central fatigue and neural influences, we intentionally used this method to bypass these factors and specifically isolate muscle fatigue development. The experimental protocol also did not facilitate evaluation of other potential mediators of muscle fatigue, such as reactive oxygen species. Lastly, the current data were collected in rats, and the direct translation to humans remains to be determined. Despite these limitations, the findings of this study remain robust and critically advance our understanding of the mechanistic relationship between muscle fatigue development and intramuscular H^+^ and Pi concentrations.

## 5 Conclusions

The current findings provide evidence that intramuscular Pi accumulation influences muscle fatigue development in vivo, as demonstrated by the discernible twitch force–Pi breakpoint and the consistent relationship between the decrease in twitch force and intramuscular Pi accumulation. In contrast, the lack of a consistent relationship between the decrease in twitch force and intramuscular H^+^ accumulation does not align with the primary mechanisms by which acidosis is thought to mediate muscle fatigue. Together, these results support the concept of a critical Pi threshold beyond which Pi mediates muscle fatigue development. Understanding the distinct roles of Pi and H^+^ in muscle fatigue has important implications for interpreting fatigue across various athletic disciplines and disease states, where altered bioenergetic and fatigue dynamics may underlie functional impairments.

## Author Contributions

**Matthew T. Lewis:** conceptualization, data curation, formal analysis, investigation, methodology, software, validation, visualization, writing – original draft, writing – review and editing. **Fabio G. Laginestra:** conceptualization, formal analysis, software, validation, visualization, writing – original draft, writing – review and editing. **Jesse C. Craig:** conceptualization, validation, visualization, writing – review and editing. **Markus Amann:** funding acquisition, resources, writing – review and editing. **Russell S. Richardson:** funding acquisition, resources, writing – review and editing. **Robert W. Wiseman:** data curation, funding acquisition, investigation, methodology, project administration, resources, software, supervision, writing – review and editing. **Ryan M. Broxterman:** conceptualization, data curation, formal analysis, funding acquisition, project administration, resources, software, supervision, validation, visualization, writing – original draft, writing – review and editing.

## Conflicts of Interest

The authors declare no conflicts of interest.

## Data Availability

The data that support the findings of this study are available from the corresponding author upon reasonable request.
